# Treatments of dental crown caries, root surface caries, and dentin hypersensitivity using the instant adhesive cyanoacrylate

**DOI:** 10.1002/ccr3.2864

**Published:** 2020-04-15

**Authors:** Hourei Oh, Naohiro Kubota, Kazuya Masuno, Yoshimasa Makita

**Affiliations:** ^1^ Department of Innovation in Dental Education Osaka Dental University Hirakata Japan; ^2^ Kubota Dental Clinic Takatsuki Japan; ^3^ Department of Chemistry Osaka Dental University Hirakata Japan

**Keywords:** caries progression suppression, cyanoacrylate, dentin hypersensitivity suppression, instant adhesive

## Abstract

The instant adhesive cyanoacrylate is appropriate for the suppression of caries progression, dentin hypersensitivity, and secondary caries.

## Graphical abstract

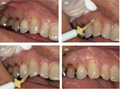

## 1 INTRODUCTION

Caries is a globally endemic disease. The FDI World Dental Federation proposed a concept that will shift the global trend of minimal intervention from dental treatment centered on tooth conservation to that based on preventative care. Accordingly, we studied the efficacy of the instant adhesive cyanoacrylate to treat dental crown caries, root surface caries, and dentin hypersensitivity.

In α‐cyanoacrylate, which is the primary ingredient of instant adhesives, a hydrogen molecule that bonds with the carbon on the α‐position of the acrylate is replaced by a CN base. In other words, its structural formula is CH_2_ = C(CN)COOR. In 1949, the industrial production method of the monomer was developed by Goodrich Inc.^1^ However, as there was no demand, it was mostly ignored. In 1955, while synthetic fibers were being studied by Eastman Chemicals in the USA, it was accidentally discovered that α‐cyanoacrylate has superior glass bonding capacity. Furthermore, it became evident from the results of repeated studies that the substance remaining after removal of the so‐called inactive resins, such as polyethylene and Teflon, from this compound could adhere strongly and instantly. This led to the introduction of cyanoacrylate adhesives worldwide.^2^

Subsequently, studies on the application of instant adhesives in medicine began in the 1960s.^3^ For example, in the Vietnam War, the CA spray was used to suppress bleeding in injured soldiers until they could be admitted to a hospital. In addition, it was used to suture skin, blood vessels etc, during surgical procedures. In the field of veterinary medicine, it was used to restore bone, skin, and tortoise shells.^4^

Even in Japan, the use of cyanoacrylate in the 1960s in small intraoral surgeries and the conservation and hemostasis of wounds resulting from tooth extraction, endodontic treatment etc, has been reported.^5^, ^6^, ^7^

In instant adhesives, the cyanoacrylate monomer, which is the main component, becomes hardened rapidly in response to water on the surface of the bonding materials and polymerizes into a polymer. When put together, bonding materials adhere strongly.^8^ This instant adhesive has the advantages of quick action at ordinary temperatures, being a single‐component solvent‐free substance, and good transparency when cured. As it has high adhesive capacity, and its safety for use in the living body and oral cavity has been confirmed from its clinical application over approximately half a century since its introduction, its potential for arresting caries progression and improving the symptoms of dentin hypersensitivity has been hypothesized. It has been reported that 38% silver diammine fluoride is effective for the suppression of primary caries, secondary caries, and hypersensitivity to dentin. However, the lesions of the tooth became black, and the esthetics were extremely impaired.^9^ In this study, we applied cyanoacrylate, which was used as a transparent and aesthetic medical instant adhesive, for the effective treatment of crown caries, root caries, and dentin hypersensitivity.

## 2 CASE REPORT

The instant medical adhesive α‐cyanoacrylate was applied at the affected site in a 58‐year‐old man, whose general condition was good, in the three cases described below. In this case study, the patient's right to privacy was respected, and informed consent was obtained.

**Figure 1:**
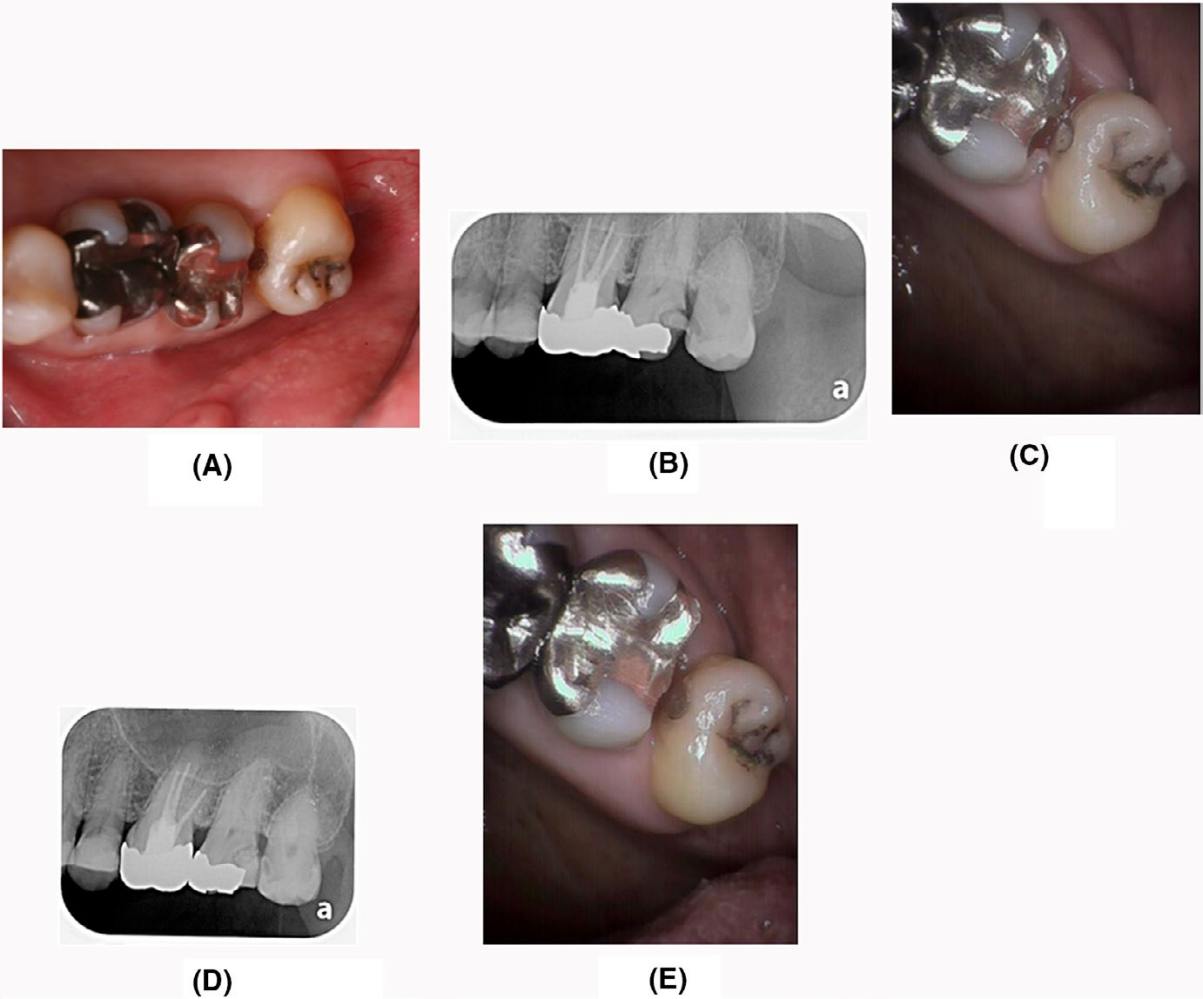
Efficacy for smooth surface caries of the tooth crown. First examination: smooth surface caries on the mesial proximal surface of the maxillary left third molar. Symptoms: No spontaneous pain was observed. Pain was experienced during food impaction. The patient's general condition was good. A, Photograph of the caries site in the oral cavity during the first examination. B, X‐ray image during the first examination. C: Photograph after the application of cyanoacrylate. D, X‐ray image immediately after the application of cyanoacrylate. E, Photograph of the oral cavity 15 days after the application of cyanoacrylate. F, X‐ray image of the oral cavity 15 days after the application of cyanoacrylate. Treatment progress: No pain was observed during the first examination. The patient complained of pain during food impaction. Cyanoacrylate was applied. No subjective symptoms 15 d after the application, and no pain even when food fragment pressure was present. An X‐ray image also confirmed no progression of caries

In the first case, the efficacy of α‐cyanoacrylate for smooth surface caries of the tooth crown when applied to the affected site was examined. Visual examination, probing, and X‐ray imaging confirmed smooth surface caries on the mesial surface of the maxillary left third molar (Figure 1A). Caries of the affected site progressed up to the dentine, and probing showed that the areas were slightly soft. Cold water and heat did not induce pain. There was mild pain during food impaction, and the presence of cavities was confirmed via X‐ray imaging (Figure 1B). The affected site was dried, and foreign bodies, such as food fragments, were removed before applying cyanoacrylate (Figure 1C, D). After the application, the patient experienced no discomfort, such as pain. However, 15 days later, the patient experienced spontaneous pain or pain owing to food impaction. The affected site became hardened, showing progression inhibition (Figure 1E). X‐ray imaging showed no changes, thereby confirming progression suppression (Figure 1F).

**Figure 2:**
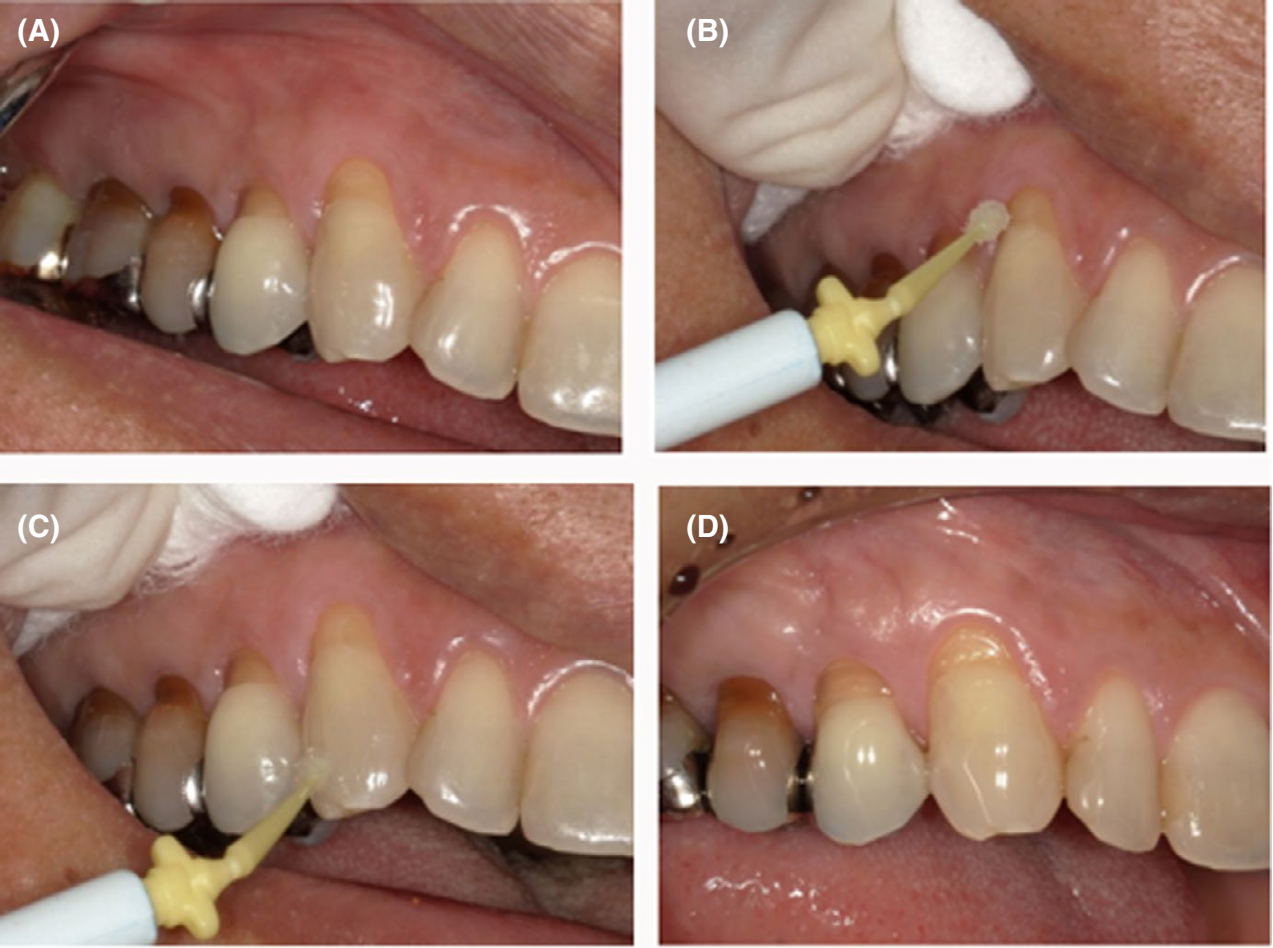
Efficacy for dentin hypersensitivity. First examination: hypersensitivity of the maxillary right canine, confirmed via visual inspection and probing. Symptoms: No spontaneous pain was observed. Pain induced by abrasion, cold air, cold water, and hypersensitivity was observed. The general condition of the patient was good. A, Photograph of the oral cavity during the first examination. B, Before application to the affected site. C, Immediately after the application. D, Thirty days after the application. Treatment progress: No spontaneous pain was observed during the first examination. Pain was observed when the tooth was exposed to cold air. Cyanoacrylate was applied, and 30 d later, no subjective symptoms were observed. Pain induced by abrasion, cold air, and cold water improved. There were no abnormal findings in tissues surrounding the tooth, such as the gingiva

In the second case, the efficacy of α‐cyanoacrylate for dentin hypersensitivity was examined on the labial surface of the maxillary right canine, confirmed via visual inspection and probing (Figure 2A). There was no spontaneous pain. Cracks were observed on the enamel surface. Pain induced by abrasion, cold air, cold water, and hypersensitivity was observed. Cyanoacrylate was applied to the surface of the affected site (Figure 2B). Subsequently, no more pain was observed (Figure 2C). Thirty days later, the symptoms had subsided (Figure 2D). Moreover, there were no signs of inflammation in the tissues surrounding the tooth.

**Figure 3:**
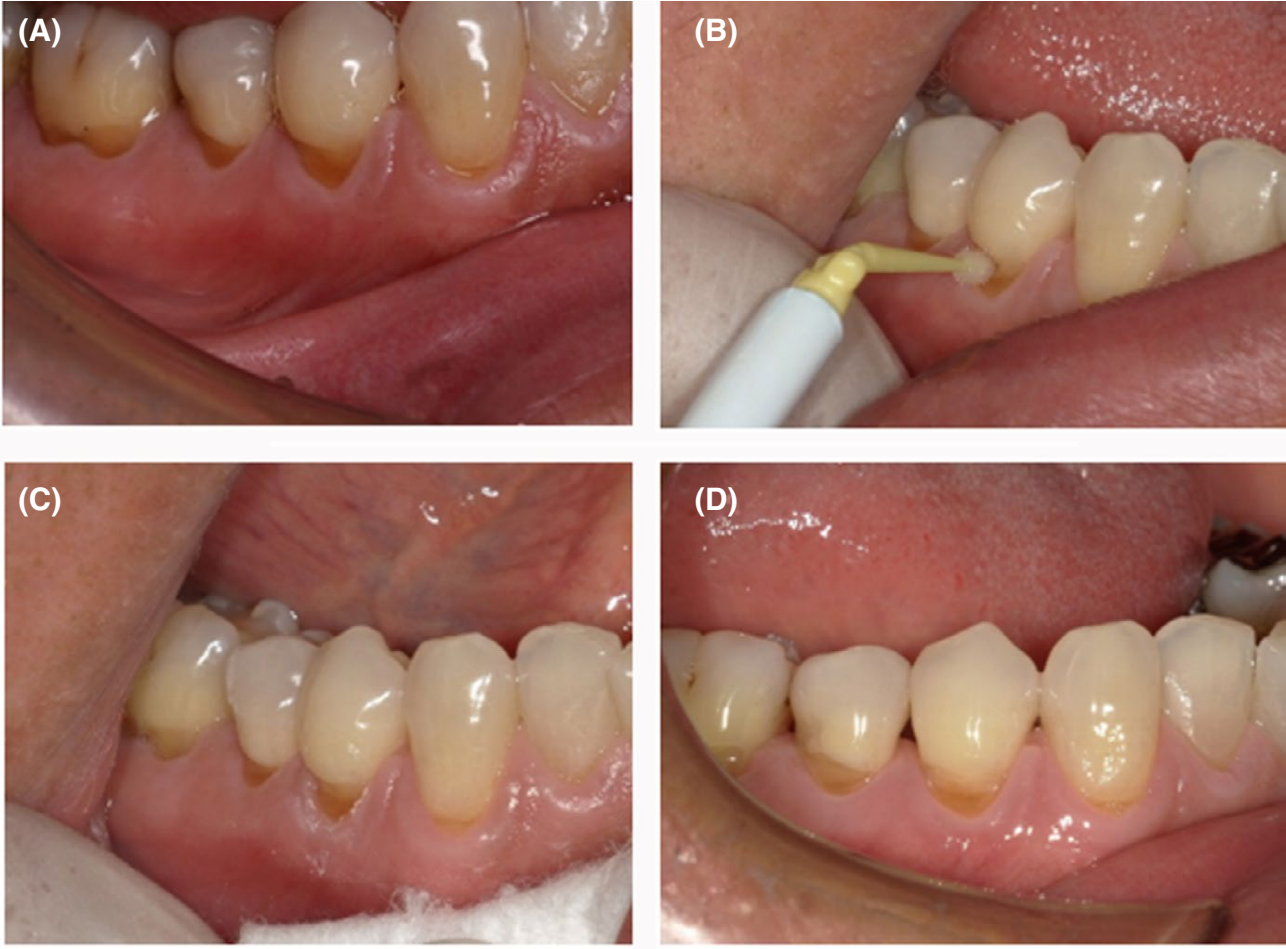
Efficacy for root surface caries. First examination: Root surface caries present in the neck of the mandibular right first premolar. Symptoms: No spontaneous pain was observed. Excessive pain was observed while brushing teeth. The patient's general condition was good. A, Photograph of the oral cavity during the first examination. B, Affected site during the application. C, After the application. D, Thirty days after the application

In the third case, the efficacy of α‐cyanoacrylate for root surface caries was examined (Figure 3A). The affected area was confirmed via visual examination and probing. Root surface caries was observed in the neck of the mandibular right first premolar (Figure 3B). There was no spontaneous pain. A typical wedge defect was present on the tooth neck, and the patient experienced excessive pain while brushing teeth. With probing, the affected area was observed to be soft, and slight activity was judged. Cyanoacrylate was applied to the affected site (Figure 3C), and 30 days later, the symptoms had subsided (Figure 3D).

Treatment progress: No spontaneous pain was experienced during the initial examination. The patient experienced excessive pain while brushing teeth. Cyanoacrylate was applied, and subsequently, no subjective symptoms were observed for 30 days. The excessive pain owing to tooth brushing also improved. No abnormal findings were observed in tissues surrounding the tooth, such as the gingiva.

## 3 DISCUSSION

In these case studies, progression suppression of dental crown caries, root surface caries, and dentin hypersensitivity could be confirmed with the use of the instant medical adhesive α‐cyanoacrylate.

Tooth structures and teeth lost owing to caries are restored using various types of materials to recover tooth function and esthetics. As the oral cavity is constantly moist and subjected to repeated stress owing to mastication, there is a demand for materials with various properties to restore function in the oral cavity safely over the long term. One of various restorative materials appropriate for this purpose can be selected.

Cyanoacrylate has a chemical adhesive mechanism with tooth substance. It has a different mechanism of adhesion than other dental cements. In other words, it has a high affinity for basic substances, such as amines, and it becomes hardened through the anionic polymerization of the calcium ions in the natural tooth dentin by the cyano and carbonyl groups present in cyanoacrylate.^10^ Moisture is present in minute ducts of the surface of natural tooth dentin. With glass ionomer cement and carboxylate cement, metal ions that do not react with polyacrylate, ions that react with polyacrylate, freshly generated calcium polyacrylate etc, are in the soluble state; therefore, once they come in contact with water, the generated substance dissolves, forming a sturdy matrix. Therefore, the adhesive strength decreases in the presence of moisture. With cyanoacrylate liners, which have a different adhesive mechanism, the hardening reaction progresses with moisture as a catalyst. Therefore, moisture present on the dentine surface does not reduce the adhesive strength. Compared with other dental cements, cyanoacrylate can achieve stronger adhesion. Furthermore, the hardening mechanism of cyanoacrylate, which is richer in moisture compared with enamel, enables instant and solid adhesion with dentine, which has more organic substances.^11^ In addition, formaldehyde production owing to degradation is low in cyanoacrylate; therefore, it is safe.^10^

The cyanoacrylate monomer, which is the main component of instant adhesives, becomes hardened rapidly as it reacts with water on the surface of the bonding material. Subsequently, it polymerizes to become a polymer that adheres strongly to similar bonding materials. This instant adhesive has the advantages of quick action at ordinary temperatures, being a one‐component solvent‐free substance, and good transparency of the hardened substance. Therefore, it has high adhesive strength. This adhesion mechanism consists of three steps: mechanical bonding, physical interaction, and chemical interaction.^8^ Mechanical bonding is also called an anchoring effect; the liquid adhesive enters the pores on the material surface, where it becomes hardened to establish adhesion. This explains the adhesion of materials with absorption capacity, such as wood, fiber, and skin. Physical interaction is a force between molecules (van der Waals force); having a secondary bonding force is the fundamental principle of adhesives. Chemical interaction is referred to as the primary bonding strength; thus, a covalent bond and hydrogen bond, which are expected to have the strongest adhesive strength, are formed. As such, “adhesion” is established via mechanical grappling, intermolecular force, and interatomic force, and thus, it is complex.^12^

When caries is present, it either progresses, arrests, or regresses. Therefore, progression suppression of caries involves three situations: caries progresses but slowly; caries is arrested, and caries recovers. In the first case, after arresting the progression of caries in the adjacent surface for a certain period, cutting was performed; eventually, a filler was considered necessary. In the second case, where dentin hypersensitivity was present, the progression and expansion of caries could only be arrested with cutting intervention. Moreover, it is limited to cases where functionality and esthetics can be recovered only by cutting. Clinically, dentin hypersensitivity refers to sharp pain induced by mechanical and chemical stimulation of the dentine surface.^13^ Direct causes that have been reported are the abrasion of the tooth neck, caries, loss of the wedge shape, acid erosion, reduction deformities of the enamel quality, deterioration of the enamel, exposure of the tooth root dentine owing to involution of the gingiva after periodontal treatment, and induction related to occlusion. Hydrodynamic theory is widely considered the onset mechanism of dentin hypersensitivity.^14^ Therefore, commercially available suppressants of dentin hypersensitivity are those that mobilize fluid into dentinal tubules by coagulating proteins in them and those that either cover or close open dentinal tubules. Cyanoacrylate is believed to either cover or close dentinal tubules. The causal factors of dentin hypersensitivity are several, as are the treatment methods. Therefore, improvement in symptoms when an instant adhesive is applied to the affected site is considered efficacious. The root caries in the third case was located in well‐defined tarnished softened areas; therefore, a probe could be easily inserted. During the removal, there was slight resistance. The affected area was defined as the area limited to the cement enamel boundary or the roots.^15^ Generally, the activity of the affected area is judged based on palpation. When the affected area is hard, it is judged to be “nonactive or active”; when it is soft, it is judged to be “active.” This is likely effective for active or nonactive root surface caries, as in this study. Moreover, cyanoacrylate has been reported to show inhibitory effects on the growth of *Streptococcus, Neisseria, Catarrhalis, Gaffkya,* and *Staphylococcus aureus* in vitro, demonstrating its antibacterial action ^16^ ^.^ Cyanoacrylate likely displays its prevention and antibacterial action in areas affected by caries and enamel quality through strong adhesion to the exposed dentine.

Minimal intervention (dental treatment with minimal invasion) is a concept where preventive intervention on teeth changes the global flow toward common dental treatments.^17^ We expect that cyanoacrylate has a similar effect to 38% silver diammine fluoride, which suppresses the progress of caries without repairing. However, 38% silver diammine fluoride has the disadvantage of changing the color of tooth to black. In contrast, cyanoacrylate, which is transparent and has excellent esthetics, overcomes this disadvantage.^18^

This method suppressed the progression of pain and caries. However, it is only a temporary treatment, and we think that conservative and prosthetic treatments are needed after this method. It could be effective for the caries of the deciduous teeth.

## 4 CONCLUSION

The instant adhesive cyanoacrylate is appropriate for the suppression of caries progression, dentin hypersensitivity, and secondary caries. In the future, studies on the indications, time of application, frequency of application etc, in a larger number of patients are required.

## CONFLICT OF INTEREST

None declared.

## AUTHOR CONTRIBUTIONS

All the authors were involved in study interpretation and writing and reviewing the manuscript, including final approval. Additionally, HO and NK were involved in study development.
